# Iraq and Afghanistan War Veterans with Reintegration Problems: Differences by Veterans Affairs Healthcare User Status

**DOI:** 10.1007/s10488-014-0564-2

**Published:** 2014-06-11

**Authors:** Nina A. Sayer, Robert J. Orazem, Siamak Noorbaloochi, Amy Gravely, Patricia Frazier, Kathleen F. Carlson, Paula P. Schnurr, Heather Oleson

**Affiliations:** 1 Center for Chronic Disease Outcomes Research, Minneapolis VA Healthcare System, One Veterans Drive, Minneapolis, MN 55417 USA; 2 Department of Medicine, University of Minnesota, Minneapolis, MN USA; 3 Departments of Psychiatry, University of Minnesota, Minneapolis, MN USA; 4 Department of Psychology, University of Minnesota, Minneapolis, MN USA; 5 Center to Improve Veteran Involvement in Care, Portland VA Medical Center, Portland, OR USA; 6 National Center for PTSD, White River Junction, VT USA; 7 Geisel School of Medicine at Dartmouth, Hanover, NH USA; 8 Department of Public Health and Preventive Medicine, Oregon Health and Science University, Portland, OR USA

**Keywords:** Veterans, Healthcare service needs, Mental health, Posttraumatic stress disorder, Traumatic brain injury, Department of Veterans Affairs Healthcare

## Abstract

We studied 1,292 Iraq and Afghanistan War veterans who participated in a clinical trial of expressive writing to estimate the prevalence of perceived reintegration difficulty and compare Veterans Affairs (VA) healthcare users to nonusers in terms of demographic and clinical characteristics. About half of participants perceived reintegration difficulty. VA users and nonusers differed in age and military background. Levels of mental and physical problems were higher in VA users. In multivariate analysis, military service variables and probable traumatic brain injury independently predicted VA use. Findings demonstrate the importance of research comparing VA users to nonusers to understand veteran healthcare needs.

## Introduction

United States (US) combat operations in Afghanistan, Iraq and neighboring countries, referred to as Operation Enduring Freedom (OEF), Operation Iraqi Freedom (OIF), and Operation New Dawn (OND), taken together comprise the longest war the US has fought since the Vietnam War and the first extended war to depend on an all-volunteer military. Identifying and treating the needs of service members and veterans who served in these operations is a high priority for both the Department of Defense (DoD) and the Department of Veterans Affairs (VA). It is also a priority for clinicians outside of these two Federal healthcare systems who provide services to veterans, but may not have expertise on deployment-related health concerns or easy access to resources for combat veterans.

Research indicates that US service members returning from the wars in Iraq and Afghanistan carry a high burden of mental health disorders, with posttraumatic stress disorder (PTSD) being particularly common (Hoge et al. 2004; Hoge et al. 2006; Milliken et al. 2007; Smith et al. 2008). A 2010 systematic review reported that PTSD prevalence estimates ranged between 10.3 and 17 % in studies based on surveys of combat troops formerly deployed to Iraq (Sundin et al. 2010). The wars in Iraq and Afghanistan have also heightened concern about the sequelae of traumatic brain injury (TBI), which some have designated the “signature injury” of these wars because of its prevalence relative to prior wars (DePalma et al. 2005; Okie 2006). Findings based on surveys of individuals formerly deployed to Afghanistan and Iraq suggest that 10–23 % may have had a deployment-related TBI (Sayer et al. 2014). Furthermore, with one notable exception (Schell and Marshall 2008), research indicates that the prevalence of reported mental health concerns and TBI increases as time since deployment increases (Milliken et al. 2007; Polusny et al. 2011; Sundin et al. 2010). Indeed, it is likely that Iraq and Afghanistan war veterans will have elevated healthcare needs long after US military operations in these countries cease.

Importantly, even in the absence of a diagnosed deployment-related health condition, individuals formerly deployed to combat zones may have difficulty transitioning from military to civilian roles. In a national survey of OEF/OIF veterans who use VA healthcare, Sayer et al. (2010) found that at least 25 % were having some to extreme difficulty in major life domains after their deployments including social functioning, productivity, community involvement and self-care. Although these problems were more common in those with probable PTSD, high proportions of the OEF/OIF veterans faced challenges in multiple domains of functioning and community involvement regardless of their mental health status (Sayer et al. 2010). Policy makers and providers need a better understanding of not only the mental and physical health burdens but also the functional problems in veterans returning from war to design strategies to promote their full and productive participation in civilian life.

Studies describing postdeployment healthcare needs of those formerly deployed to Iraq and Afghanistan have been based primarily on active duty service members who access the military health system (Hoge et al. 2004, 2006; Milliken et al. 2007; Smith et al. 2008) or veterans seeking VA healthcare (Sayer et al. 2010; Scholten et al.^2012^; Seal et al. 2009, 2010; Taylor et al. 2012). Although this research is informative, it leaves unanswered questions concerning the health and postdeployment reintegration of OEF/OIF/OND veterans who do not make use of VA healthcare after their military discharge. Under the National Defense Authorization Act of 2008, all OEF/OIF/OND veterans are eligible for five years of free VA healthcare for any condition possibly related to combat service (U.S. Department of Veterans Affairs 2011). However, not all OEF/OIF/OND veterans have enrolled in and used the VA. At the time of this writing, 58 % of eligible Iraq and Afghanistan veterans had used VA healthcare since their military discharge (U.S. Department of Veterans Affairs, Epidemiology Program 2014), compared with roughly 30 % of Vietnam and 25 % of Korean and WWII veterans (U.S. Department of Veterans Affairs 2010a).

Research based on veterans of prior wars has identified meaningful differences between veterans who used VA healthcare and those who did not. For example, it has been reported that veterans who used VA were more likely to be nonwhite, of lower socioeconomic status, and to have a higher prevalence of disability, major chronic conditions, and obesity than veterans who did not use VA (Agha et al. 2000; Hynes et al. 2007; Koepsell et al. 2009; Littman et al. 2012; Nelson et al. 2007). It is unknown whether these patterns of differences in sociodemographics and disease burden extend to OEF/OIF/OND veterans. OEF/OIF/OND veterans have enrolled in VA for healthcare at a much higher rate than veterans from prior service eras, probably because of large-scale outreach efforts accompanying the National Defense Authorization Act of 2008. Because of this, there may be fewer differences between OEF/OIF/OND veterans who enroll in VA and those who do not. If there are fewer differences, then research based on VA samples of OEF/OIF/OND veterans may generalize to those who do not use VA healthcare. Regardless, an understanding of the postdeployment healthcare needs of VA users and nonusers is necessary to inform service delivery for those who receive healthcare within and outside of the VA healthcare system.

This study makes use of data collected as part of a large, national randomized clinical trial (RCT) of expressive writing in OEF/OIF/OND veterans with self-reported reintegration difficulty. We focused on veterans who believed that they had reintegration difficulty because they would be more likely to also be interested in receiving help to adjust to civilian life. Our first objective was to estimate the prevalence of perceived overall reintegration difficulty in the entire population of OEF/OIF/OND veterans. The remaining two objectives focused only on veterans who believed they were having reintegration difficulty. Specifically, our second objective was to compare sociodemographic characteristics and the mental and physical health of OEF/OIF/OND veterans with perceived reintegration difficulty who used to those who do not use VA healthcare. Our third objective was to identify predictors of VA healthcare user status among veterans with perceived reintegration difficulty.

## Methods

### Data Source

This study used variables from the DoD Defense Manpower Data Center (DMDC) roster of all OEF/OIF/OND veterans who had left active duty military service, and assessment data from a RCT of expressive writing in OEF/OIF/OND veterans with perceived reintegration difficulty. This study was approved by the Minneapolis VA Healthcare System and University of Minnesota institutional review boards and U.S. Army Medical Research and Materiel Command Human Research Protection Office.

### Participants

**Fig. 1:**
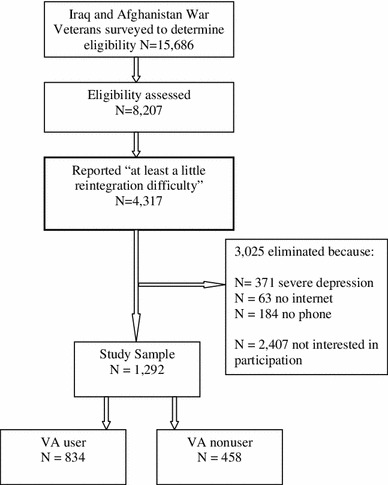
Pathway from recruitment to study sample

Figure 1 displays the pathway from our recruitment pool to inclusion in our study. We recruited the sample of veterans from the DMDC roster, which included 1,396,477 veterans at the time of this study. We randomly selected a gender-stratified sample of 20,000 veterans. We oversampled women, who comprised 12 % of the population, such that they comprised 30 % of our potential recruitment pool.

We used mail survey methods that involved repeat mailing to nonresponders and a $5 monetary incentive (Dillman 2000) to assess inclusion and exclusion criteria in our recruitment pool. We stopped recruitment after contacting 15,686 veterans because we had reached our target sample size. Inclusion criteria were: self-report of “at least a little difficulty readjusting back into civilian life” assessed with a general single item used in prior research (Sayer et al. 2010); internet access; and providing an email address and telephone number. We used subjective report of overall reintegration difficulty because we were interested in identifying a sample who perceived a need for help with reintegration problems regardless of whether these problems were objectively clinically significant. In preliminary research, we found that veterans who reported at least “a little difficulty” reintegrating on this single item had poorer mental health and more functional difficulties than those who reported “no difficulty” reintegrating into community life. Exclusion criteria included severe depression as identified by the patient health questionnaire-eight-item depression scale (PHQ-8; Kroenke et al. 2009). We excluded individuals with severe depression because prior studies found that those with severe depression did not benefit from the intervention tested in our RCT (Baikie et al. 2012; Kovac and Range 2002).We also asked veterans if they would be interested in participating in our online expressive writing intervention and invited those who were eligible and interested to participate.

Of the 15,686 veterans contacted (our effective recruitment pool), 8,207 (52 %) returned our eligibility survey within the study timeframe, 4,317 (53 %) of whom reported “at least a little difficulty readjusting back into civilian life” on the general single item used to assess study eligibility that was described above. Among those with any self-reported reintegration difficulty, 618 (14 %), including 371 (9 %) who screened positive for severe depression, did not meet our other inclusion/exclusion criteria and 2,407 (56 %) were not interested in participating in our online RCT as indicated by either their response to our survey question assessing interest or nonresponse to our invitation to participate. Almost two-thirds (*n* = 829; 64 %) of the 1,292 veterans included in our sample had sought VA healthcare according to the DMDC roster.

## Measures

DMDC roster variables were available for veterans in our recruitment pool. All the other measures listed below were available for the 1,292 veterans included in our RCT.

### DMDC Roster Variables

We used the following DMDC roster variables: gender, race, military branch, military component, time since discharge, and VA user status.

### Other Sociodemographic Characteristics

We assessed age, education, employment and student status, income, marital and parental status, service connection, and military rank. We also obtained more detailed information regarding race and ethnicity than was available in the DMDC roster.

### Trauma History

We assessed lifetime trauma history using the posttraumatic stress diagnostic scale (PDS; Foa 1995) and combat exposure using the combat experiences scale from the Deployment Risk and Resilience Inventory (DRRI; King et al. 2003; Vogt et al. 2008).

### Psychological Distress

We measured psychological distress with the Brief Symptom Inventory (BSI-18; Derogatis 2000) which contains 18 items that assess depression, anxiety, and somatization and together yield a global severity index. Participants indicated the degree to which each symptom caused them distress in the past 2 weeks on a 5-point scale ranging from 0 (*not at all*) to 4 (*extremely*). Total scores, which are formed by averaging the scores across the items, range from 0 to 4, with higher scores indicating greater distress. Internal consistency (Cronbach’s alpha) in the study sample was 0.93.

### Anger

The BSI Hostility subscale, a separate 5-item subscale from the BSI, is a measure of anger and anger expression. Response format and scoring rules were the same as those employed for the BSI-18. The hostility subscale, like the other BSI scales, has strong psychometric properties (Derogatis 1993). Cronbach’s alpha in the study sample was 0.77.

### Physical Symptoms

The Pennebaker Inventory of Limbic Languidness (PILL; Pennebaker, 1982) is a 54-item scale that assesses the frequency of occurrence of common physical symptoms and sensations. In this study we removed two PILL items that overlapped with BSI somatization items and one that was highly redundant with another PILL item so that our measure consisted of 51 items. In addition, for ease of administration as part of this assessment battery, we used the same time frame and response format for the PILL that we used for the BSI. Total scores were determined by counting the number of symptoms that were rated “2” (“moderately” distressing or bothersome) or higher. Thus, total scores ranged from 0 to 51, with higher scores indicating endorsement of a greater number of physical symptoms at moderate to extreme levels. Cronbach’s alpha in our sample was 0.95.

### Probable PTSD

We assessed probable PTSD using the PTSD checklist-military version (PCL-M; Weathers et al. 1995). The PCL-M consists of the 17 DSM-IV PTSD symptoms that are rated on a 5-point Likert scale ranging from 1 (*not at all*) to 5 (*extremely*). Consistent with DSM-IV criteria for PTSD, we defined probable PTSD cases as those who reported over the past month at least one symptom of moderate or greater severity from cluster B (reexperiencing symptoms), three symptoms of moderate or greater severity from cluster C (avoidance symptoms), and two symptoms of moderate or greater severity from cluster D (hyperarousal symptoms).

### Probable Traumatic Brain Injury (TBI)

Those who indicated on the Combat Experiences Scale that they were injured or wounded in combat were administered the first two questions from the Brief TBI Screen (Schwab et al. 2007). Probable TBI was defined as endorsement of both: (1) “During any of your deployments, were you injured from any of the following: fragment/shrapnel wound above the shoulder, bullet wound above the shoulder, vehicular accident or crash (any type of vehicle, including airplane), fall, blast/explosion (improvised explosive device, RPG, land mine, grenade, mortar, artillery, etc.), other type of blow to the head”, and (2) “Did any injury you received while deployed result in any of the following immediately afterwards: Being dazed, confused, or “seeing stars”; not remembering the event; losing consciousness; head injury or concussion.”

### Reintegration Difficulty

We used the Military to Civilian Questionnaire (M2C-Q) to assess past month reintegration difficulty (Sayer et al. 2011). The M2C-Q has 16-items that assess difficulty in the following reintegration domains: social relations (8 items), productivity (e.g., schooling, employment; domestic life; 3 items), community or civic engagement (2 items), perceived meaning in life (1 item), and self-care and leisure (2 items). Responses range from 0 (*no difficulty*) to 4 (*extreme difficulty*); four items (e.g., parenting problems) also offer a “not applicable” response option. Total scores were formed by summing across items and dividing by the number of completed items, with higher scores indicating greater reintegration difficulty. Initial research supports the construct and factor validity of scale scores (Sayer et al. 2011). Cronbach’s alpha in this sample was 0.92.

### Social Support

Perceived social support over the past month was assessed with the Post-Deployment Social Support Scale from the DRRI (King et al. 2003). This scale measures the extent to which family, friends, coworkers, employers, and the community provide emotional and instrumental support. Each of the 15 items is scored on a 5-point scale with responses ranging from 1 = “strongly agree” to 5 = “strongly disagree.” Total scores were formed by adding across items. The scale scores have high internal consistency and associations with mental health outcomes support construct validity (Vogt et al. 2008). Cronbach’s alpha in the study sample was 0.85.

### Life Satisfaction

Global life satisfaction over the past month was measured with the satisfaction with life scale (SWLS; Diener et al. 1985). The five items were rated on a 7-point scale ranging from 1 = “strongly disagree” to 7 = “strongly agree”. Two-month test–retest reliability has been found to be 0.82. The one factor structure of the SWLS has been established via exploratory factor analysis and the validity of the SWLS scores have been found to exceed other comparable measures of well-being (Frazier et al. 2003). Cronbach’s alpha in the study sample was 0.92.

### Binge Drinking and Tobacco Use

The following questions were used to assess binge drinking: (1) “Have you had any alcohol in the past month?”; (2) “Over the past month, how many days per week did you have a drink containing alcohol on average?”; and (3) “Over the past month, how many times did you have 5 (for men)/4 (for women) or more drinks on one occasion?” These questions were taken from the Behavioral Risk Factor Surveillance System (BRFSS) survey (Naimi et al. 2003) with the one modification being that we defined binge drinking differently for men than for women because of gender differences in gastric metabolism of alcohol (Wechsler et al. 1995). Binge drinking was defined as consumption of 4 or 5 or more drinks on at least one occasion in the past 30 days for women and men, respectively. In addition, we asked participants how many cigarettes per day they smoked on average over the past month.

### Health Services Use

We asked participants to indicate the number of times they have seen a medical professional (doctor, nurse, psychologist, psychiatrist, social worker, rehabilitation specialist of any kind, etc.) for a physical illness or injury or a medical professional (doctor, nurse, psychologist, psychiatrist, social worker, etc) for a mental health concern in the past 3 months. Responses were dichotomized to represent any physical or mental healthcare use in the past 3 months.

### Data Analysis

This was a stratified random sample with oversampling of women. Therefore, we derived all estimates based on weighted analyses. Weights were generated to reflect the inverse probability of participants from specific subpopulations being sampled.

To estimate the prevalence of perceived reintegration difficulty in the population of Iraq and Afghanistan veterans (Objective 1), we stratified the recruitment pool (*N* = 15,686) into four groups based on gender (male, female) and VA user status (yes, no). We then constructed a stratified logistic regression model of perceived reintegration difficulty (yes, no) among the 8,207 responders to our eligibility survey based on the DMDC roster variables. This model was used to predict perceived reintegration difficulty (yes, no) among the 7,479 eligibility survey nonresponders. To increase the precision of our estimate of perceived reintegration difficulty beyond our recruitment pool, we divided each gender by user group into 96 strata based on the other four variables included in the DMDC roster and projected these proportions to the population based on the sizes of these strata in the entire population of OEF/OIF/OND veterans.

Objectives 2 and 3 focused on the subgroup of Iraq and Afghanistan veterans with perceived reintegration difficulty. To extrapolate from study measures based on the 1,292 included in our RCT to the subpopulation of OEF/OIF/OND veterans with perceived reintegration difficulty, we modeled two forms of potential bias: (a) nonresponse to our eligibility survey, and (b) among survey responders with perceived reintegration difficulty, nonparticipation in our RCT. We used logistic regression to estimate the propensity of both forms of potential bias based on DMDC roster variables available for our recruitment pool. Specifically, for nonresponse adjustment, we constructed 9 propensity classes per gender: 3 based on the estimated propensity of nonresponse to the eligibility survey, and within these 3 strata, an additional 3 based on the estimated propensity of nonparticipation in the RCT. All subsequent analyses adjusted for these propensity strata and then weighted to the population of those with perceived reintegration difficulty.

Objective 2 focused on sociodemographic and clinical differences between VA users and nonusers. To address objective 2, we tested for differences in demographic characteristics between VA users and nonusers using stratified Chi square tests and stratified regression. Next, we tested for differences in clinical characteristics between VA users and nonusers while controlling for observed demographic differences using stratified logistic regression with adjustment based on propensity analysis.

Objective 3 involved identifying unique predictors of VA user status. To address this objective, we used multivariate stratified logistic regression. We included in these models only those variables that were significantly associated with VA user status in bivariate analyses at *P* < 0.05 (see Tables 1 and 2).

**Table 1:** Sociodemographic characteristics of Iraq and Afghanistan war veterans with perceived reintegration difficulty by VA user status

	Population		VA nonuser		VA user	
Characteristic	Weighted proportion or mean	SE	Weighted proportion or mean	SE	Weighted proportion or mean	SE
Age >40 years (%)***	28.95	0.90	23.43	1.43	31.87	1.16
Female (%)	10.78	0.003	10.92	0.003	10.68	0.003
Race (%)						
White only	65.68	1.64	66.77	2.98	65.10	1.94
Black only	11.75	1.05	10.82	1.85	12.24	1.28
Asian only	3.28	0.64	2.71	1.10	3.58	0.79
Native American only	1.57	0.43	1.10	0.69	1.83	0.55
Multiracial/other	4.22	0.73	5.24	1.50	3.68	0.77
Not reported	13.51	1.23	13.37	2.29	13.58	1.44
Hispanic ethnicity (%)	15.82	1.31	15.45	2.41	16.01	1.54
Marital status (%)						
Never married/single	23.93	1.51	25.05	2.81	23.34	1.77
Married/partnered	60.13	1.65	59.92	2.95	60.24	1.98
Divorced/separated	15.61	1.26	14.78	2.27	16.04	1.51
Widowed	0.33	0.18	0.25	0.21	0.37	0.25
Has one or more children (%)	62.80	1.61	59.24	2.80	64.68	1.96
Education (%)						
High school diploma or GED	12.22	1.23	13.46	2.33	11.57	1.42
Some college	46.23	1.70	49.55	3.08	44.48	2.03
College diploma	30.53	1.53	27.18	2.67	32.31	1.86
Advanced degree	9.14	0.77	8.77	1.14	9.34	1.01
Other	1.87	0.41	1.04	0.44	2.31	0.58
Student past 3 months (%)	34.74	1.66	36.47	3.03	33.83	1.96
Working past 3 months (%)	76.67	1.45	79.78	2.52	75.03	1.77
Income (%)						
$10,000 or less	6.29	0.91	6.09	1.66	6.40	1.07
$10,001 to $20,000	11.74	1.19	12.58	2.28	11.30	1.36
$20,001 to $40,000	23.53	1.51	21.27	2.66	24.72	1.82
$40,001 to $60,000	21.32	1.44	19.02	2.49	22.53	1.76
More than $60,000	28.45	1.31	32.12	2.35	26.52	1.58
Prefer not to answer	8.67	0.99	8.93	1.91	8.53	1.12
Service branch (%)***						
Army	59.88	1.64	51.59	3.05	64.27	1.92
Marines	18.22	1.40	23.95	2.77	15.18	1.56
Navy	11.86	0.91	11.91	1.58	11.83	1.11
Air force	10.04	0.76	12.55	1.36	8.71	0.92
Military rank (%)						
Enlisted	88.61	0.94	87.48	1.65	89.21	1.14
Warrant officer	1.00	0.30	0.75	0.37	1.13	0.42
Officer	10.39	0.90	11.78	1.63	9.65	1.08
Military component (%)						
Active duty	58.29	1.69	56.75	3.07	59.11	2.01
Reserves/national guard	38.29	1.67	40.00	3.04	37.38	1.98
Other^a^	3.42	0.57	3.25	0.95	3.51	0.70
Years since deployment (*M*)**	5.85	0.08	5.55	0.15	6.01	0.09
Service connected for mental health condition (%)***	19.51	1.30	6.55	1.45	26.38	1.84
Service connected for physical health condition (%)***	46.54	1.56	22.34	2.40	59.38	2.02

*VA* Department of Veterans Affairs, *GED* general equivalency diploma

* *p* < 0.05; ** *p* < 0.01; *** *p* < 0.001

^a^Other included inactive ready reserve, civilian or government employee, and other

**Table 2:** Clinical characteristics of Iraq and Afghanistan war veterans with perceived reintegration difficulty by VA user status

	Population		VA nonuser		VA user	
Characteristic	Weighted proportion or mean	SE	Weighted proportion or mean	SE	Weighted proportion or mean	SE
Trauma exposure (*M*)	3.68	0.07	3.56	0.12	3.74	0.09
Combat exposure (*M*)***	5.89	0.14	5.16	0.25	6.28	0.18
Probable TBI (%)***	10.07	1.05	3.19	1.13	13.71	1.49
Probable PTSD (%)**	34.95	1.66	27.88	2.88	38.69	2.03
Distress (*M*)**	1.04	0.03	0.94	0.05	1.09	0.03
Anger (*M*)	1.16	0.03	1.11	0.05	1.20	0.03
Physical symptoms (*M*)**	10.55	0.32	9.19	0.55	11.25	0.40
Social support (*M*)	53.31	0.37	54.29	0.70	52.79	0.43
Life satisfaction (*M*)	20.74	0.28	21.33	0.50	20.43	0.33
Reintegration difficulty (*M*)*	1.41	0.03	1.31	0.06	1.46	0.04
Any alcohol use past month (%)	75.07	1.48	76.29	2.64	74.43	1.78
Binge drinking past month (%)	50.41	1.73	49.87	3.16	50.70	2.05
Daily cigarette use past month (%)						
None	75.72	1.55	75.65	2.83	75.75	1.83
20 or fewer	20.24	1.46	20.68	2.69	20.01	1.73
21 or more	4.04	0.72	3.67	1.26	4.24	0.89
Any mental health clinic visits in past 3 months (%)***	26.51	1.48	15.85	2.19	32.16	1.95
Any physical health clinic visits in past 3 months (%)***	47.35	1.68	38.56	2.91	52.00	2.06

*VA* Department of Veterans Affairs, *TBI* traumatic brain injury, *PTSD* posttraumatic stress disorder

* *p* < 0.05; ** *p* < 0.01; *** *p* < 0.001

All tables include weighted estimates. We used SAS 9.3 for all computations.

## Results

### Reintegration Difficulty in OEF/OIF/OND Veterans

The estimated prevalence of at least a little reintegration difficulty in the OEF/OIF/OND population was 54 % (95 % CI 0.53–0.55). The estimated prevalence of reintegration difficulty was higher among VA users (62 %; 95 % CI 61–63 %) than among those who did not use VA healthcare (45 %; 95 % CI 44–47 %) (*p* < 0.0001).

### Sociodemographic Characteristics by VA User Status

Table 1 displays sociodemographic characteristics in the population of OEF/OIF/OND veterans with perceived reintegration difficulty and differences by VA user status. A larger proportion of veterans who used VA healthcare were 40 years or older. A larger proportion of VA users had served in the Army, whereas a larger proportion of nonusers had served in the Marines or Air Force. VA users had on average been discharged from military service for 6 months longer than nonusers. Additionally, a larger proportion of VA users reported receiving VA benefits for military-related (referred to as “service connected”) mental and physical health conditions.

### Clinical Characteristics by VA User Status

Table 2 displays clinical characteristics in the population of OEF/OIF/OND veterans with perceived reintegration difficulty and differences by VA user status. There was no difference in terms of lifetime trauma history. However, VA users reported higher levels of combat exposure. Perhaps not surprisingly based on this fact, VA users were also more likely to meet study criteria for probable TBI and PTSD. They also reported higher levels of psychological distress, physical symptoms, and reintegration difficulty. There were no differences in anger, perceived social support, or life satisfaction. Proportions of alcohol use, binge drinking and smoking were similarly high across groups. The differences in combat exposure, prevalence of probable PTSD, prevalence of probable TBI, distress, physical symptoms, and reintegration difficulty between VA users and VA nonusers remained significant when we adjusted for differences in age, military branch, and time since deployment (data not shown). Perhaps reflecting these clinical differences between VA users and nonusers, VA users were much more likely to report having sought medical care for physical or mental health problems over the past three months.

### Predictors of VA User Status

Table 3 presents results from multivariate analysis evaluating the unique predictors of VA user status among veterans with perceived reintegration difficulty. As can be seen, service branch, service-connected mental and physical health conditions, time since deployment, and probable TBI were independent predictors of VA user status. Those who served in the Army were more than twice as likely to be VA users than those who served in the Air Force or Marines. As time since deployment increased, the likelihood of being a VA user increased. Those with service- connected mental and physical health conditions were also two to four times more likely to be VA users. Last, veterans with probable TBI were more than twice as likely to be VA users.

**Table 3:** Multivariate analysis of association between veteran characteristics and use of department of Veterans affairs healthcare among veterans with perceived reintegration difficulty

Variable	Adjusted odds ratio	95 % CI
Age >40 years	1.11	0.83–1.48
Service branch		
Army vs. air Force***	2.32	1.51–3.57
Air force vs. navy	0.65	0.39–1.11
Air force vs. marines	1.00	0.57–1.75
Army vs. navy	1.52	0.97–2.37
Army vs. marines***	2.33	1.46–3.71
Navy vs. marines	1.53	0.87–2.69
Years since deployment*	1.08	1.01–1.15
Service connected for mental health condition**	2.40	1.36–4.24
Service connected for physical health condition****	4.10	2.85–5.91
Probable TBI*	2.66	1.16–6.11
Probable PTSD	1.43	0.85–2.38
Distress	1.13	0.74–1.72
Physical symptoms	0.98	0.96–1.01
Reintegration difficulty	0.93	0.70–1.22

*CI* confidence interval, *TBI* traumatic brain injury, *PTSD* posttraumatic stress disorder

* *p* < 0.05; ** *p* < 0.01; *** *p* < 0.001; **** *p* < 0.0001

## Discussion

We estimate that about half of Iraq and Afghanistan veterans perceived at least a little reintegration difficulty and therefore might have felt the need for help readjusting to civilian life. Interestingly, on average almost 6 years had passed since military discharge among veterans with perceived reintegration difficulty. This indicates that reintegration problems are not transient in some veterans and, therefore, may not resolve without intervention.

Unlike studies based on samples from prior war eras (Agha et al. 2000; Hynes et al. 2007; Koepsell et al. 2009; Littman et al. 2012; Nelson et al. 2007), we did not find that VA users differed from nonusers in terms of minority or indicators of socioeconomic status. The fact that employment levels were similar across groups suggests that lack of employer-based health insurance may be less of a factor in the decision to use VA healthcare among this newest generation of veterans than among veterans of prior military service eras. Instead, we found that factors related to military service, including military service branch, time since discharge, and combat exposure were associated with VA user status. We find it interesting that military branch was associated with VA user status. It may be that VA’s outreach efforts are stronger in the Army than in other branches of the military or that military culture differs by branch and therefore that outreach messages need to be tailored specifically for Marines and Air Force troops. The finding that service-connected disability predicted VA user status was consistent with prior research (Hynes et al. 2007). This is not surprising given that veterans with service-related disabilities have the highest priority for VA healthcare and may also quality for reduced or no copays. Also consistent with prior studies that had identified poorer health status in VA users (Agha et al. 2000; Koepsell et al. 2009; Littman et al. 2012), we found that VA users had greater mental and physical health burden than VA nonusers. In particular, OEF/OIF/OND veterans with perceived reintegration difficulty who had used VA healthcare were more likely to have probable TBI and PTSD, and reported higher levels of psychological distress, physical symptoms, and reintegration difficulty. Military service-related variables and probable TBI were independent predictors of VA user status in the multivariate model.

The VA has considerable expertise in and dedicated resources for the assessment and treatment of deployment-related problems, including TBI and PTSD. Our findings lend further support for the importance of maintaining this capacity. Importantly, however, although many of the assessed clinical problems were more prevalent in VA users, they were not absent in nonusers. The possible unmet healthcare needs of veterans with deployment-related difficulties who do not use VA healthcare is a significant public health concern. In fact, the prevalence of probable PTSD exceeded 25 % in VA nonusers with at least a little perceived reintegration difficulty. Although not necessarily related to deployment, the high prevalence of binge drinking in VA users and nonusers also warrants concern. Thus, providers in the private sector should be aware of postdeployment and other common health problems in returning veterans and be prepared to help them access the VA healthcare system if they do not have the expertise to address these difficulties in their practice setting.

Taken together, our results suggest that findings from research based on samples of Iraq and Afghanistan veterans who use VA may not generalize to veterans who do not use VA services. The same was true of research based on veterans from prior military service eras. Overall, there remain significant gaps in our understanding of the healthcare needs of Iraq and Afghanistan veterans who do not seek VA healthcare because most research on veterans is conducted by VA researchers studying VA users. Indeed, this study underscores the importance of conducting research on VA nonusers to obtain a more complete picture of the healthcare needs of US veterans and to help ensure that all veterans have access to needed services.

## Limitations

We used DMDC roster variables to estimate the prevalence of perceived reintegration difficulty in the population of OEF/OIF/OND veterans based on responses to our eligibility survey. Unfortunately, we did not have other variables for all veterans included in our recruitment pool. Furthermore, our method for extrapolating from eligibility survey responders to nonresponders was based on the assumption that the relationship between perceived reintegration difficulty and DMDC roster variables was the same in both groups. Although we believe it to be reasonable, this assumption could not be verified. A related limitation is that we used DMDC roster variables to adjust for potential bias associated with nonresponse to our survey and nonparticipation in our RCT among those who perceived reintegration difficulty. It is, however, likely that there were other differences between eligibility survey nonresponders and responders and between veterans who did not participate and those who did participate in our RCT for which we were not able to adjust. For example, the exclusion of those with severe depression in our RCT may have resulted in lower estimates of mental and physical health symptoms than are actually present in the population of veterans with perceived reintegration difficulty. Thus, further research is needed to confirm or improve upon these estimates.

We used a single item to identify veterans who perceived themselves as having reintegration difficulty and thus might be interested in and benefit from help for readjustment issues. However, postdeployment reintegration is a complex, multi-faceted construct (Resnik et al. 2012) and we did not assess the level and types of impairments represented by those who reported “at least a little” reintegration difficulty compared to those who reported no reintegration difficulty. Another limitation was that we examined differences by VA user status among veterans with perceived reintegration difficulty rather than among the entire population of OEF/OIF/OND veterans. Thus, findings regarding differences by VA user status probably do not generalize to OEF/OIF/OND veterans who do not believe they have any reintegration problems. Another limitation is that we determined VA user status at one point in time only. However, VA user status likely changes over time and some of those classified as VA nonusers may have subsequently become VA users. A final limitation is that we did not assess some key variables related to access, including how far veterans lived from a VA, private insurance coverage and dual use of VA and other health care systems. These are variables that should be included in future studies that compare VA users and nonusers as they have implications for the planning and coordination of healthcare delivery.

## Conclusions

This study focused on veterans with perceived reintegration difficulty, which we estimate to be about half of the OEF/OIF/OND veteran population. It is the first study of which we are aware that compared OEF/OIF/OND VA users to nonusers. Most demographic differences between groups were related to veterans’ military service. There were meaningful clinical differences between groups, with greater proportions of VA users reporting military-related problems including probable PTSD and TBI and higher symptom levels than VA nonusers. However, clinical problems were not absent in VA nonusers who reported reintegration difficulty; binge drinking and probable PTSD were particularly prevalent in this group. This research confirms that findings describing health and adjustment problems in VA healthcare users cannot be generalized to VA nonusers. More research on veterans who do not use the VA for healthcare is needed to obtain a fuller picture of Iraq and Afghanistan war veterans and their healthcare needs.
